# Variations in Alcohol-Metabolizing Enzymes in People of East Indian and African Descent from Trinidad and Tobago

**Published:** 2007

**Authors:** Shelley Moore, L.K. Montane-Jaime, Lucinda G. Carr, Cindy L. Ehlers

**Keywords:** Alcoholism, alcohol dependence, alcohol disorders, Trinidad and Tobago, Indo-Trinidadians, Afro-Trinidadians, genetics and heredity, genetic polymorphisms, allele, ethnic groups, risk factors, protective factors, ethanol metabolism, alcohol dehydrogenase (ADH), aldehyde dehydrogenase (ALDH), acetaldehyde, catalase, cytochrome P4502E1 (CYP2E1)

## Abstract

The population of Trinidad and Tobago is composed mainly of people of East Indian (Indo-Trinidadians) and African (Afro-Trinidadians) ancestry. Differences in alcoholism rates exist between these two ethnic groups, and researchers have investigated whether these differences can be explained in part by variations in the genes encoding the alcohol-metabolizing enzymes alcohol dehydrogenase (ADH) 1B and 1C, and aldehyde dehydrogenase (ALDH) 1 and 2. Studies have demonstrated that a certain variant of the gene encoding ADH1B (ADH1B*3) is associated with a reduced risk of alcoholism in Afro-Trinidadians, as is a variant of the gene encoding ADH1C (i.e., ADH1C*1) in Indo-Trinidadians. An ALDH2 variant shown to have protective effects primarily in East Asians was not found in either Trinidadian ethnic group. However, a variant in the gene encoding cytosolic ALDH1A (i.e. ALDH1A1*1/*2) was found to be associated with an increase in alcohol dependence in Indo-Trinidadians.

The island of Trinidad, the southernmost island of the Lesser Antilles in the Caribbean, together with its much smaller sister island of Tobago constitute the Republic of Trinidad and Tobago, a developing country that, with the 1973 increase in oil prices, has become a relatively prosperous and industrialized nation. The population of Trinidad and Tobago is multiethnic; the two main ethnic groups are people of East Indian (Indo-Trinidadians) and African (Afro-Trinidadians) ancestry (Central Statistical Office 1990, 2003).

With the sudden economic development and industrialization that began in the 1970s, alcohol consumption levels on the islands escalated enormously. Ethnic studies investigating the prevalence of alcoholism in Trinidad and Tobago found that alcoholism prevalence is substantially higher among Indo-Trinidadians than among Afro-Trinidadians. For example, a recent assessment survey reported an alcohol problem rate of 47 percent among Indo-Trinidadians, compared with 33 percent among Afro-Trinidadians (The National Alcohol and Drug Abuse Prevention Centre 2000). However, few studies have investigated biological factors that could explain the apparent difference in alcoholism prevalence between these two ethnic groups. This article summarizes the findings of some studies analyzing genetic differences in alcohol-metabolizing enzymes between Indo- and Afro-Trinidadians that might account for some of the observed differences in alcohol dependence.

## Alcohol Metabolism and Its Role in Alcohol Dependence

Genetically influenced differences in alcohol metabolism have been implicated strongly in the etiology of alcoholism. These variations mainly result from naturally occurring variations (i.e., polymorphisms) in the genes that code for the enzymes involved in alcohol metabolism. Thus, these genes have been considered candidates that could contribute to individual differences in alcohol metabolism and, consequently, response to alcohol and vulnerability to developing alcohol dependence and alcohol-related disabilities (Crabb 1995; Li et al. 2000).

Alcohol primarily is metabolized to acetaldehyde in an oxidation reaction that is mediated (i.e., catalyzed) by enzymes known as alcohol dehydrogenases (ADHs). Other pathways of alcohol oxidation to acetaldehyde involve the enzyme catalase and the microsomal ethanol-oxidizing system (MEOS), whose key component is cytochrome P4502E1 (CYP2E1) (see Agarwal 2001). The acetaldehyde produced in all these reactions is, in turn, metabolized to acetate in a reaction catalyzed by the aldehyde dehydrogenase (ALDH) enzymes. Many of the enzymes involved in alcohol metabolism—several ADHs, CYP2E1, and ALDHs—exist in different, genetically determined forms (i.e., isoforms) that differ in their level of activity. Each person’s rate of alcohol metabolism is determined by the isoforms he or she carries. For example, in a person carrying a less active ADH or CYP2E1 isoform, alcohol is broken down at a relatively slow rate. Conversely, in a person carrying more active isoforms of ADH or CYP2E1, alcohol is broken down at a faster rate, leading to an increased formation of acetaldehyde. Finally, the presence of a less active form of ALDH results in slow acetaldehyde oxidation and the buildup of acetaldehyde in the body following alcohol consumption.

Differences in the sequences of the genes that code for these enzymes are called polymorphisms, and these polymorphisms show ethnic diversity. One of the best understood polymorphisms of alcohol-metabolizing enzymes involves the gene that encodes the enzyme ALDH2. One allele of the *ALDH2* gene, ALDH2*2, codes for a virtually inactive form of the enzyme. People carrying this allele experience alcohol-induced flushing, show an increased level of response to alcohol, and have lower rates of alcohol use and alcoholism compared with people without this allele (Wall et al. 1992). The ALDH2*2 allele is found in a large proportion (about 40 percent) of people of Far East Asian descent but is uncommon in people of other ethnicities.

## Frequencies of *ADH* and *ADH* Alleles in Indo- and Afro-Trinidadians

In searching for possible explanations for the differing alcoholism rates in Indo- and Afro-Trinidadians, researchers have examined the prevalence of various alleles of the alcohol- and acetaldehyde-metabolizing enzymes. One such study found a significant difference in the distribution of alleles of the gene that codes for a type of ADH called ADH1C. One allele of that gene, labeled ADH1C*2, encodes an ADH1C enzyme with somewhat reduced activity compared with the enzyme encoded by the ADH1C*1 allele. Montane-Jaime and colleagues (2006) found that 44 percent of Indo-Trinidadians had one ADH1C*2 and one ADH1C*1 allele (i.e., were heterozygous), and 5 percent carried two ADH1C*2 alleles (i.e., were homozygous).^1^ In contrast, only 23 percent of Afro-Trinidadians studied had one ADH1C*2 allele and only 1 percent were homozygous. The allele was significantly associated with alcohol dependence—that is, people with at least one ADH1C*2 allele were at higher risk of developing alcohol dependence than people without the allele. Finally, alcoholics with at least one ADH1C*2 allele had significantly elevated levels of certain liver enzymes (i.e., alkaline phosphatase and gamma-glutamyltransferase) that are frequently associated with heavy drinking and alcoholic liver disease. Or conversely, it appears that the ADH1C*1 allele has a protective effect, particularly in Indo-Trinidadians.

Another analysis investigated the association of alleles of another type of ADH, *ADH1B*, with alcohol dependence, drinking history, and liver function in Indo- and Afro-Trinidadians (Ehlers et al. 2007). That study determined the frequencies of two alleles, ADH1B*2 and ADH1B*3. The ADH1B*2 allele was found only in three Indo-Trinidadian participants (one alcoholic and two control subjects). However, 41 percent of the Afro-Trinidadian participants had at least one ADH1B*3 allele, and three Afro-Trinidadian participants were homozygous for the allele. Only one Indo-Trinidadian participant had at least one ADH1B*3 allele. Furthermore, people with at least one ADH1B*3 allele were significantly less likely to be alcohol dependent and had lower alcohol consumption levels compared with people without the allele. Among those participants who were alcohol dependent, ADH1B*3 was associated with significantly higher levels of the liver enzyme aspartate aminotransferase, which can be indicative of alcohol-induced liver disease. These analyses suggest that in this sample of Trinidadians, the ADH1B*3 allele has a protective effect against development of alcoholism; however, in people who do become alcohol dependent, the allele is associated with an enhanced risk for liver disease (Ehlers et al. 2007).

A third analysis of this island population sought to determine whether *ALDH* genotypes also affect alcohol dependence and liver function tests in the two major ethnic groups of Trinidad and Tobago (Moore et al. 2007). That study found that none of the subjects carried the protective ALDH2*2 allele, demonstrating that, in contrast to people of East Asian ancestry, this allele is uncommon in people of East Indian ancestry. Moreover, the findings confirm previous data that ALDH2*2 is uncommon in people of African ancestry.

However, some variation existed in the gene encoding cytosolic ALDH1 (i.e., the *ALDH1A1* gene). About 10 percent of the people studied carried one copy of the variant ALDH1A1*2 allele (i.e., had an ALDH1A1*1/*2 genotype). Of these, 4 were Afro-Trinidadians (2 alcoholics and 2 control subjects) and 20 were Indo-Trinidadians (18 alcoholics and 2 control subjects). Moreover, two articipants (one Indo-Trinidadian alcoholic and one Afro-Trinidadian alcoholic) were homozygous for ALDH1A1*2 (i.e., had the ALDH1A1*2/*2 genotype). Additionally, four participants possessed an ALDH1A1*3 allele, all of whom were Afro-Trinidadian control subjects. Indo-Trinidadian participants with at least one ALDH1A1*2 allele were more likely to be alcohol dependent. Among Afro-Trinidadians, however, the small number of subjects with atypical *ALDH1A1* polymorphisms limited any conclusions on the possible impact on alcoholism in that population (Moore et al. 2007).

## Conclusions

Several conclusions can be drawn from the studies of *ADH* and *ALDH* polymorphisms in Trinidadians. First, the presence of ADH1C*1 in Indo-Trinidadians and ADH1B*3 in Afro-Trinidadians is associated with reduced risk for alcoholism. Second, neither Indo- nor Afro-Trinidadians have the ALDH2*2 allele commonly seen in East Asians that causes flushing following alcohol consumption and protects people from developing alcoholism. Third, the presence of at least one copy of a variant in the gene encoding for cytosolic ALDH1A (i.e. ALDH1A1*2) was found to be associated with an increase in alcohol dependence in Indo-Trinidadians. Although considerable gaps in knowledge remain regarding the causes underlying differences in alcoholism prevalence between Indo- and Afro-Trinidadians, these studies together highlight the utility of evaluating risk and protective factors associated with alcohol metabolism in diverse ethnic groups.

## Footnotes

1 Every person has two copies of each gene, one inherited from the mother and one inherited from the father. If both copies are the same allele, the person is called homozygous for that allele; if the copies are of different alleles, the person is called heterozygous.
